# Comparative study of myocardial function in cases of feline hypertrophic cardiomyopathy with and without dynamic left-ventricular outflow-tract obstruction

**DOI:** 10.3389/fvets.2023.1191211

**Published:** 2023-06-22

**Authors:** Takahiro Saito, Ryohei Suzuki, Yunosuke Yuchi, Haru Fukuoka, Shuji Satomi, Takahiro Teshima, Hirotaka Matsumoto

**Affiliations:** Laboratory of Veterinary Internal Medicine, School of Veterinary Medicine, Faculty of Veterinary Science, Nippon Veterinary and Life Science University, Tokyo, Japan

**Keywords:** cat, cardiomyopathy, speckle tracking echocardiography, myocardial function, strain

## Abstract

In recent years, hypertrophic cardiomyopathy (HCM) in cats has become much more common in clinical practice due to improvements in diagnostic techniques and equipment performance. One phenotype is obstructive HCM with left ventricular (LV) outflow tract obstruction (DLVOTO). It has been reported that the presence or absence of DLVOTO does not affect long-term prognosis in cats with HCM. In this study, we evaluated and compared myocardial function in HCM-affected cats with and without DLVOTO using the two-dimensional speckle-tracking echocardiography. LV longitudinal strain of the endocardial, epicardial, and whole layer and LV circumferential strain of the epicardium were significantly decreased in all HCM-affected cats compared to healthy cats. However, these values were not significantly different between those with and without DLVOTO. In contrast, the endocardial and whole layers of LV circumferential strain were only significantly decreased in HCM-affected cats with DLVOTO compared to healthy cats. This could be attributed to the fact that the LV pressure load associated with DLVOTO affected the endocardial myocardium more in the LV endocardial layer, and that lower values of LV endocardial strain lowered the values of LV strain in the whole layer. In conclusion, our results suggest that LV myocardial function may have been more compromised in the HCM-affected cats with DLVOTO.

## 1. Introduction

Hypertrophic cardiomyopathy (HCM) in the cat is a cardiac disease that is frequently encountered in the clinical practice. HCM is characterized by myocardial hypertrophy and its phenotype is diverse. The prognosis of cats with HCM also varies from asymptomatic cases to cats with congestive heart failure even at a young age (1–5). One phenotype, systolic anterior motion of the mitral valve leaflet, is an event in which the tip of the mitral valve septal apex is pulled toward the aortic outflow tract during systole. This event involved in mitral regurgitation, as well as dynamic left-ventricular (LV) outflow-tract obstruction (DLVOTO), which results in pressure overload in the left ventricle (6). DLVOTO causes an increased left ventricular pressure, which leads to increased wall stress and myocardial ischemia (7). The presence of DLVOTO in humans aggravates the pathogenesis of HCM and is a poor prognostic factor (8); however, it has been reported that the presence or absence of DLVOTO in feline HCM does not affect the long-term prognosis (4).

Recently, two-dimensional speckle-track echocardiography (2D-STE) has been widely performed to evaluate HCM in cats and humans (9–14). 2D-STE studies in cats with HCM have shown that assessment of segmental radial and circumferential deformities in systole (12) and torsion (14), as well as diastolic deformities (12, 13), can be helpful in distinguishing cats with HCM from healthy cats.

Therefore, we hypothesized that myocardial function in HCM-affected cats with DLVOTO (HOCM) would be decreased compared to HCM-affected cats without DLVOTO (HNCM). This study aimed to analyze myocardial function using 2D-STE and compared the results between the HNCM and HOCM.

## 2. Materials and methods

A prospective cross-sectional design was used in this study. This study was conducted in accordance with our institution’s guidelines (Guidelines for the Care and Use of Animals at Nippon Veterinary and Life Science University) and was approved by the university’s ethics committee (approval number: R2-4). With prior informed consent and agreement, all cat owners participated in the study.

### 2.1. Animals

Sixty-seven client-owned cats (healthy cats: *n* = 16; HCM-affected cats: *n* = 51) were included in this study. All cats underwent a physical examination, blood pressure measurement, electrocardiogram, chest radiograph and echocardiography. The cats classified as healthy cats had no abnormal findings on these examinations. Some HCM-affected cats were already on medication. No history of medication, cardiac disease, or clinical signs were noted in the other cats. The diagnosis of HCM was made by confirming LV hypertrophy on echocardiography and excluding diseases that might cause LV hypertrophy. LV hypertrophy was defined as LV wall thickness ≥ 6 mm at end-diastole as evaluated by echocardiography. LV wall thickening was confirmed using LV short-axis images averaged over three consecutive heartbeats (15). The following previously reported criteria were used to diagnose HOCM: Peak LV outflow-tract velocity (LVOT Vmax) > 2.5 m/s and systolic anterior motion of mitral valve leaflet on B-mode (14, 16). Other cardiomyopathies were ruled out by checking for normal to near-normal LV systolic function using allometric scaling, referring to previous reports (17, 18). Cats with suspected dehydration, hypertension (Systolic blood pressure greater than 160 mmHg), or other cardiovascular or systemic disease were excluded.

### 2.2. Echocardiography

Conventional echocardiography was performed by experienced researchers using Vivid E95 echocardiography scanner and 12 MHz transducer (GE Healthcare, Tokyo, Japan). During the examination, the lead II electrocardiogram was concurrently acquired and monitored on the screen. Holding during echocardiography was performed with an assisting human hand, and all cats were not sedated. Data were obtained for at least 5 heartbeats. Analysis of the echocardiographic data was performed on a separate day from the acquisition of the images by one researcher using an offline workstation (EchoPAC PC, version 204, GE Healthcare, Tokyo, Japan). The left atrial-to-aortic diameter ratio was measured in the right parasternal short-axis view at the level of basal heart. The end-diastolic interventricular septal thickness (IVSd), LV end-diastolic posterior wall thickness (LVPWd), LV end-diastolic internal diameter (LVIDd), LV end-systolic internal diameter, and fractional shortening were measured in the right parasternal short-axis view at the level of the chordae tendineae. Relative LV wall thickness (RWT) was calculated using the following formula:

RWT=(IVSd[mm]+LVPWd[mm])/(LVIDd[mm])

Trans-mitral inflow in the left apical four-chamber view was measured by pulsed-wave Doppler, and the peak velocities of the early diastolic wave (E-wave) and late diastolic wave (A-wave) were determined. The E-wave to A-wave velocity ratio (E/A) was also calculated. In the case of fusion of E- and A-waves, values for cases containing these waves were excluded.

### 2.3. Two-dimensional speckle-tracking echocardiography

An overview of the 2D-STE analysis for cats has been previously described (9, 14, 15, 19, 20). 2D-STE analysis is performed using high-quality images obtained from conventional echocardiography. For evaluation of LV myocardial deformation, images of the left ventricle at the level of the papillary muscle were acquired in the right parasternal short-axis view and the left apical four-chamber view. Left apical four-chamber view images modified for right heart measurements were also obtained to analyze right myocardial deformations (21). Longitudinal and circumferential systolic strain peaks (SL and SC, respectively) were obtained in the endocardium, the entire layer, and the epicardium of the LV (LV-SL and LV-SC, respectively) (21). SC was measured at the papillary muscle level of the LV (14, 22). The endocardial-to-epicardial strain ratio (Endo/Epi), which is believed to reflect compensatory mechanisms of cardiac function in patients with HCM, was also calculated (23, 24). Previous studies have shown observer variability in 2D-STE analysis in our laboratory (14, 19–21).

### 2.4. Statistical analysis

Variables in each table are expressed as mean ± standard deviation values. Commercially available software (R 2.8.1; https://www.r-project.org/) was used for statistical analyses. One-way analysis of variance followed by Tukey’s multiple comparison test for normally distributed data or Kruskal–Wallis test followed by Steel–Dwass test for non-normally distributed data was used to compare continuous variables between groups. The significance level was set at *p* < 0.05.

## 3. Results

### 3.1. Demographic data

Data regarding demographic characteristics and physical examination results are summarized in Table 1. Nineteen of the 51 cats with HCM belonged to the HNCM, while the remaining 32 belonged to the HOCM. There were no significant differences in age, weight, heart rate, or blood pressure between the two groups. There were significant differences in LVOT Vmax between healthy cats and the HNCM, healthy cats and the HOCM, and the HNCM and HOCM.

**Table 1:** Clinical characteristics in healthy cats and cats with cardiomyopathy.

Variables	Healthy cats (*n* = 16)	HNCM (*n* = 19)	HOCM (*n* = 32)
Age (year)	6.7 ± 4.4	6.2 ± 3.6	3.6 ± 2.7
Sex (male/female)	9/7	11/8	19/13
Body weight (kg)	4.4 ± 1.6	4.2 ± 0.7	4.0 ± 1.3
Heart rate (bpm)	200 ± 32.0	183.1 ± 37.3	182.6 ± 24.7
Systolic blood pressure (mmHg)	136.4 ± 14.3	133.3 ± 15.2	130.0 ± 16.4
LVOT Vmax (m/s)	0.9 ± 0.3	1.0 ± 0.3^*^	4.0 ± 0.9^*†^
ACVIM (B1, B2, C)	–	12, 4, 3	21, 7, 4
Medication (yes/no)	0/16 (0%)	15/19 (79%)	9/32 (28%)
ACE inhibitor	–	5/19 (26%)	3/32 (9%)
Beta Blocker	–	11/19 (58%)	5/32 (16%)
Pimobendan	–	2/19 (11%)	1/32 (3%)
Diuretic	–	1/19 (5%)	2/32 (6%)

LVOT Vmax, peak velocity of left-ventricular outflow-tract; ACVIM, American College of Veterinary Internal Medicine. Continuous variables are displayed as mean ± standard deviation. ^*^: Values that was significantly different from the healthy cats (*p* < 0.05). ^†^: Values that was significantly different from the HNCM (*p* < 0.05).

### 3.2. Echocardiography

The echocardiographic variable data are summarized in Table 2. IVSd, LVPWd, and RWT were significantly higher in the HNCM and HOCM than in healthy cats (all *p* < 0.01). E-wave velocity of the HOCM was increased compared to healthy cats and HNCM (*p* < 0.05). E-wave and E/A analyses were partially excluded because of waveform fusion in some cases.

**Table 2:** Results of conventional echocardiographic indices in cats.

Variables	Healthy cats (*n* = 16)	HNCM (*n* = 19)	HOCM (*n* = 32)
LA/Ao	1.3 ± 0.2	1.4 ± 0.4	1.4 ± 0.3
IVSd (mm)	3.8 ± 0.4	4.8 ± 1.8^*^	6.2 ± 1.5^*^
LVPWd (mm)	4.1 ± 0.7	5.3 ± 1.8^*^	6.2 ± 1.9^*^
LVIDd (mm)	14.5 ± 1.5	11.8 ± 4.6	13.3 ± 1.8
RWT	0.5 ± 0.1	0.8 ± 0.1^*^	1.0 ± 0.3^*^
FS (%)	47.5 ± 7.6	42.3 ± 6.9	48.6 ± 11.6
E-wave (m/s)	0.6 ± 0.2	0.6 ± 0.1	0.9 ± 0.3^*†^
E/A	0.1 ± 0.2	1.2 ± 0.8	1.2 ± 1.1

LA/Ao, left atrial-to-aortic diameter ratio; IVSd, end-diastolic interventricular septal thickness; LVPWd, end-diastolic LV free-wall thickness; LVIDd, end-diastolic LV internal diameter; RWT, relative left ventricular wall thickness; FS, fractional shortening; E/A, E-wave to A-wave ratio. Continuous variables are displayed as mean ± standard deviation. ^*^: The value is significantly different from the healthy cats (*p* < 0.05). ^†^: The value is significantly different from the HNCM (*p* < 0.05).

### 3.3. Two-dimensional speckle-tracking echocardiography

**Figure 1:**
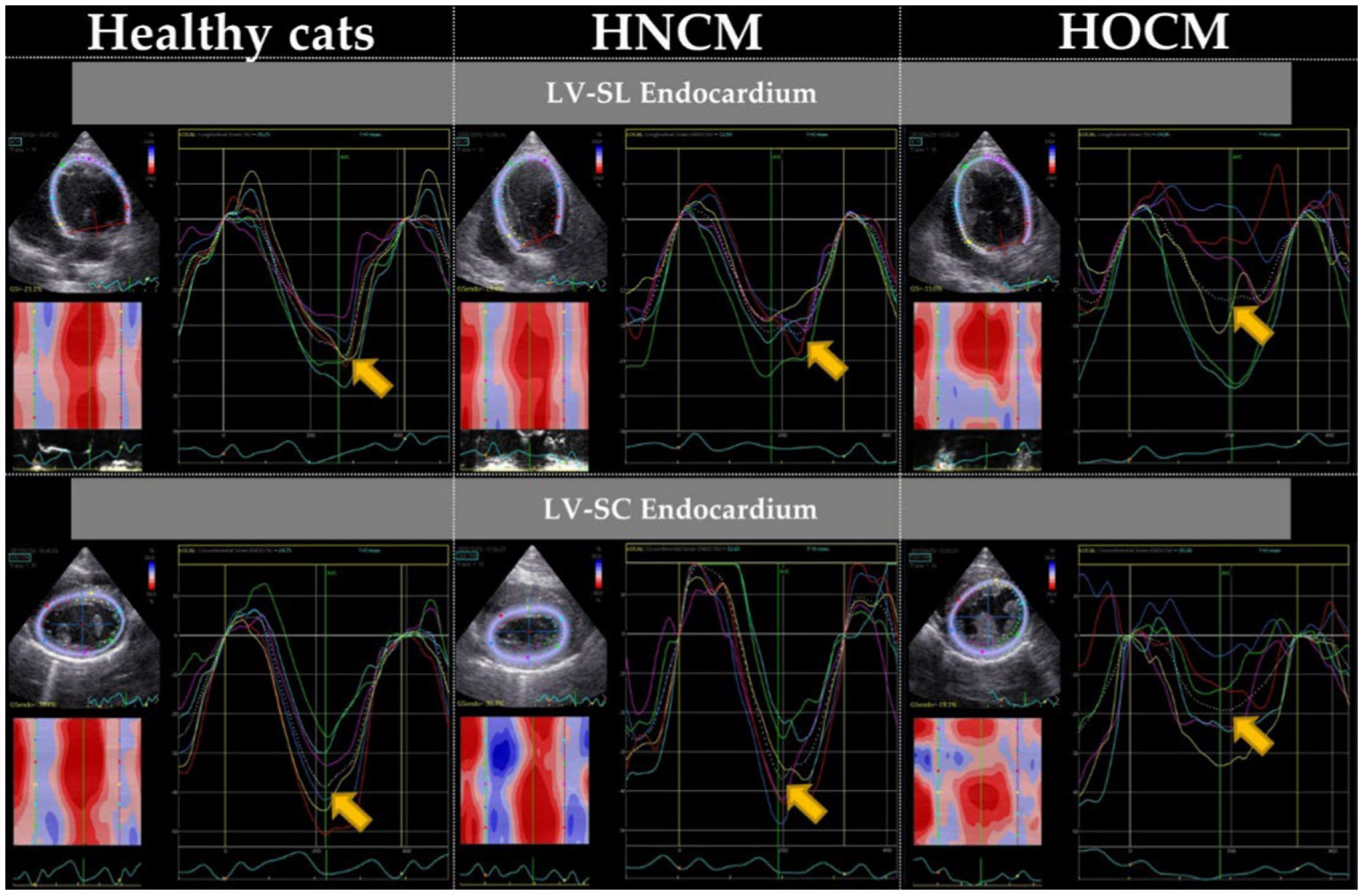
Myocardial motion analysis results of endocardium by 2D-STE method (representative data). LV-SL, peak systolic strain in the longitudinal direction at left ventricles; LV-SC, peak systolic strain in the circumferential direction at left ventricles. Arrows indicate the peak of the endocardium.

**Figure 2:**
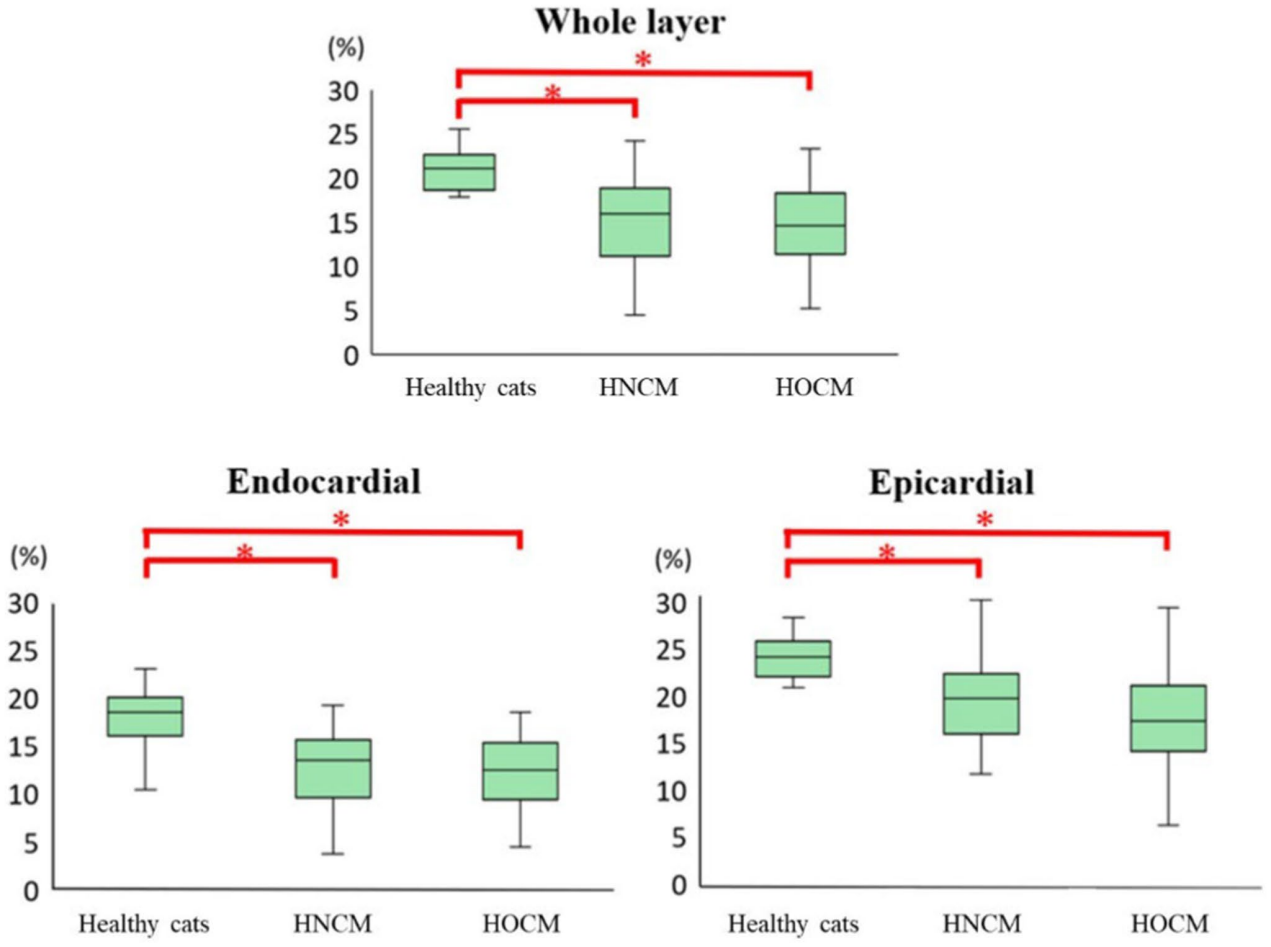
Results of longitudinal strain by 2D-STE method (box-and-whisker diagram). ^*^The value is significantly different from the healthy cats (*p* < 0.05).

**Figure 3:**
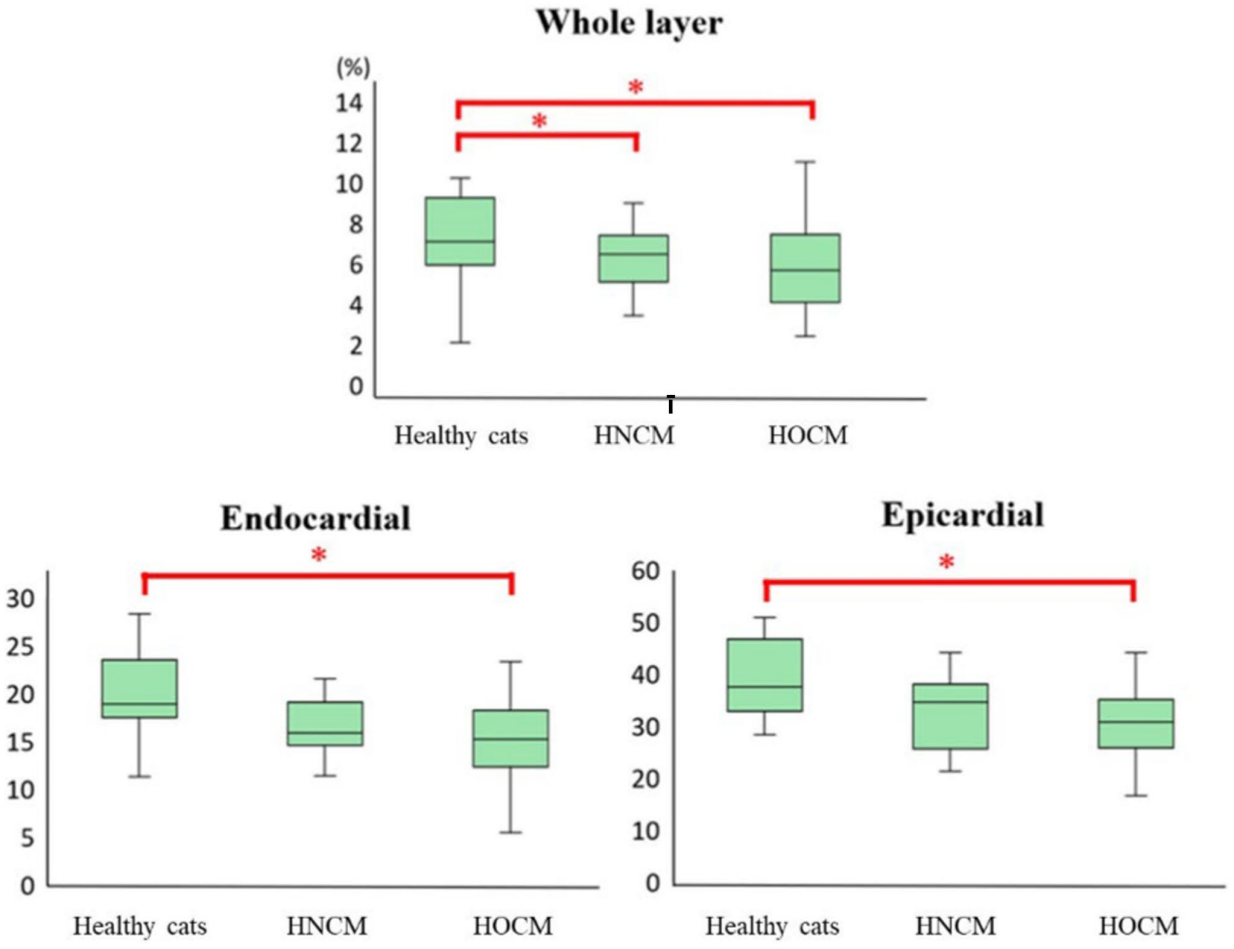
Results of circumferential strain by 2D-STE method (box-and-whisker diagram). ^*^The value is significantly different from the healthy cats (*p* < 0.05).

The 2D-STE results are summarized in Table 3. Representative results of the 2DSTE method in the endocardium are shown in Figure 1. In addition, box-and-whisker diagrams of the LV-SL and LV-SC are also shown in Figures 2, 3, respectively. The LV-SL of whole layer was significantly decreased in the HNCM and HOCM compared to healthy cats (*p* < 0.05). There was no significant difference in LV-SL in any layer between the HNCM and HOCM. LV-SC in the epicardium was significantly decreased in the HNCM and HOCM compared to healthy cats (both *p* < 0.01). LV-SC in the HOCM of the endocardium and whole layers were decreased compared to healthy cats (*p* = 0.01, *p* < 0.01 respectively). There was no significant difference in LV-SC in any layer between the HNCM and HOCM. The LV-SL Endo/Epi was increased in the HNCM and HOCM compared to healthy cats (*p* < 0.01, *p* = 0.02 respectively). In comparison, there was no significant difference in LV-SC Endo/Epi between the HNCM and HOCM.

**Table 3:** Strain assessed by two-dimensional speckle tracking echocardiography in healthy cats and cats with cardiomyopathy.

Variables	Healthy cats (*n* = 16)	HNCM (*n* = 19)	HOCM (*n* = 32)
LV-SL (%)			
Whole layer	20.3 ± 3.5	14.8 ± 5.1^*^	14.7 ± 4.3^*^
Endocardium	23.3 ± 3.8	18.9 ± 5.5^*^	18.1 ± 4.9^*^
Epicardium	17.6 ± 3.3	12.7 ± 3.7^*^	12.3 ± 3.7^*^
Endo/Epi	1.3 ± 0.1	1.5 ± 0.1^*^	1.5 ± 0.3^*^
LV-SC (%)			
Whole layer	20.1 ± 4.3	16.5 ± 2.8	15.6 ± 4.2^*^
Endocardium	38.7 ± 7.3	32.8 ± 6.2	30.6 ± 7.8^*^
Epicardium	7.4 ± 3.0	6.3 ± 1.5^*^	6.0 ± 2.4^*^
Endo/Epi	6.4 ± 3.8	5.4 ± 1.8	5.7 ± 2.3

LV-SL, peak systolic strain in the longitudinal direction at left ventricles; LV-SC, peak systolic strain in the circumferential direction at left ventricles; End, endocardium; Epi, epicardium. Continuous variables are displayed as mean ± standard deviation. ^*^: The value is significantly different from the healthy cats (*p* < 0.05).

## 4. Discussion

In this study, the HNCM and HOCM had decreased LV-SL in whole layers and LV-SC in the epicardium, which is consistent with the results of previous studies (20). Previous 2D-STE studies in cats with HCM have reported lower longitudinal strain in asymptomatic HCM cats than in healthy cats (13, 19). This has been shown to be caused by myocardial dysfunction and histopathologic changes (10, 11, 25, 26). Along with histopathological changes such as modifications in myocardial fiber orientation, myocardial compensatory mechanisms are believed to be linked with these dysfunctions (14, 19, 27). Even in human HCM, there are cases in which LV-SL in whole layers is decreased, even if the left ventricular ejection fraction appears normal. These cases have been reported to have a poor prognosis (28) and should not be judged as having normal LV systolic function based on conventional echocardiography only. However, in this study, there was no significant difference in LV-SL between the HNCM and HOCM in whole layer, so it was unlikely that the HOCM would have a worse prognosis based on LV-SL results. Previous studies on cats reported no differences in survival between the HNCM and HOCM (4). In contrast, in humans, HOCM is reported to be associated with a shorter survival time and worse prognosis owing to DLVOTO than HNCM (7). The detailed cause of why DLVOTO has a poorer prognosis in HCM is unknown. However, the mechanism has been discussed in previous papers as follows (7); The increased left ventricular pressure due to DLVOTO is expected to result in increased wall stress. Myocardium also devotes more effort to contraction, which can lead to myocardial ischemia. These abnormalities may contribute to eventual cardiomyocyte death and scarring (29–31). These changes are thought to result in stiffening of the myocardium, leading to diastolic dysfunction (32–34) as well as increased susceptibility to electrical instability and sudden death. DLVOTO of HCM-affected cats may reduce endocardial myocardial function through a mechanism similar to that reported in humans (7). In addition, the decreased LV-SC in the endocardial may have inevitably decreased LV-SC in the whole layer. The aforementioned human study also reported that older patients with HOCM (age ≥ 40 years) were more likely to experience deterioration than younger patients with HOCM by a similar mechanism (7). It is unclear whether it is appropriate to extrapolate this mechanism directly to cats, and the fact that the duration of obstruction is different in cats than in humans may be related to the finding that survival is not affected in the former. Nevertheless, based on this mechanism and the results of this study, we believe that careful evaluation of the long-term prognosis of cats with HOCM is warranted in the future.

In this study, LV-SL Endo/Epi was significantly increased in the HOCM and HNCM compared to the healthy cats, but there was no significant difference in LV-SC Endo/Epi. However, LV-SC Endo/Epi was decreased in the HOCM and HNCM compared to the healthy cats, although the difference was not significant. LV-SC Endo/Epi in asymptomatic cats-affected HCM was increased compared to healthy cats in a previous study, although there was no significant difference in LV-SL Endo/Epi (20). Increased LV-SC Endo/Epi is considered to be circumferential endocardial compensation for depressed epicardial contraction (20, 23, 24). Therefore, the HCM-affected cats included in this study were relatively severe and the compensatory mechanisms may have become dysfunctional. Although this study included cats with HCM of various American College of Veterinary Internal Medicine stages and thus the results should be interpreted with caution, a lower LV-SC Endo/Epi may be associated with worse prognosis in cats-affected HCM.

There were several limitations to the study. The sample size was small, which may have affected the results owing to case bias. In addition, some affected cats were prescribed oral medications, which may have affected the results. Furthermore, diagnosis was based on echocardiography, and not pathological findings. The study also included cats with HCM of varying severities, which may have affected the results. Specifically, it is possible that pathological severity was milder in the HNCM, resulting in a significant difference only in the HOCM compared to the healthy cats. However, no significant differences were found between the HNCM and HOCM with respect to left ventricular wall thickness or American College of Veterinary Internal Medicine stage. Therefore, it is considered that there is no extreme difference in severity of disease.

## 5. Conclusion

Our results also indicate that 2D-STE is useful for detecting HCM, as shown in previous studies, and that cats with HOCM may have worse myocardial function than those with HNCM. However, predicting prognosis requires caution, and further studies are warranted to clarify these aspects.

## Data availability statement

The original contributions presented in the study are included in the article/supplementary material, further inquiries can be directed to the corresponding author.

## Ethics statement

The animal study was reviewed and approved by Nippon Veterinary and Life Science University. Written informed consent was obtained from the owners for the participation of their animals in this study.

## Author contributions

RS and TS conceptualized and designed the study, acquired and interpreted the data, and drafted and critically revised the manuscript. YY, HF, and SS acquired, analyzed, and summarized the data and approved the article. TT and HM acquired and interpreted the data, supervised the study, and approved the article. All authors contributed to the article and approved the submitted version.

## Funding

This research was funded by the Japan Society for the Promotion of Science KAKENHI (grant number 22K05995).

## Conflict of interest

The authors declare that the research was conducted in the absence of any commercial or financial relationships that could be construed as a potential conflict of interest.

## Publisher’s note

All claims expressed in this article are solely those of the authors and do not necessarily represent those of their affiliated organizations, or those of the publisher, the editors and the reviewers. Any product that may be evaluated in this article, or claim that may be made by its manufacturer, is not guaranteed or endorsed by the publisher.

## Glossary

2D-STE: Two-dimensional speckle-tracking echocardiography
A-wave: Peak velocity of the late diastolic wave
DLVOTO: Dynamic left-ventricular outflow-tract obstruction
E-wave: Peak velocity of the early diastolic wave
E/A: E-wave to A-wave velocity ratio
FS: Fractional shortening
HCM: Hypertrophic cardiomyopathy
HNCM: Non-obstructive HCM
HOCM: Obstructive HCM
IVSd: End-diastolic interventricular septal thickness
LA/Ao: Left atrium to aortic diameter ratio
LV: Left-ventricular
LVIDd: End-diastolic LV internal diameter
LVOT Vmax: Peak velocity of left-ventricular outflow-tract
LVPWd: End-diastolic LV free-wall thickness
RWT: Relative left-ventricular wall thickness
SC: Circumferential strain
SL: Longitudinal strain
